# Primary Malignant Melanoma of the Esophagus With Unusual Endoscopic Findings: A Case Report and Literature Review

**DOI:** 10.1097/MD.0000000000003479

**Published:** 2016-04-29

**Authors:** Hui Liu, Yan Yan, Chun-Meng Jiang

**Affiliations:** From the Department of Gastroenterology, The Second Affiliated Hospital of Dalian Medical University, Dalian, Liaoning, China.

## Abstract

Primary malignant melanoma of the esophagus (PMME) is a rare disease with an extremely poor prognosis. We experienced a 79-year-old man with PMME who had unusual endoscopic findings. On endoscopy, an elongated lump was detected on 1 side of the vertical axis of the esophagus. The mass extended progressively for 15 cm along the esophageal longitudinal axis and invaded half of the esophageal circumference. These endoscopic findings were not characteristic of PMME, and the condition was confirmed with biopsy and immunohistochemical staining. Here, we present this rare case and review the recent relevant literature regarding PMME. Doctors should be aware that PMME might present with unusual endoscopic findings.

## INTRODUCTION

Primary malignant melanoma of the esophagus (PMME) is an extremely rare but highly aggressive tumor that accounts for 0.1% to 0.5% of all primary esophageal malignancies.^1^ The mean survival time from diagnosis is only 13.4 months, and the 5-year survival rate is 4.2%.^1,2^ A definitive diagnosis may be obtained with pathological analysis and identification of positivity for S-100 and human melanoma black (HMB)-45 on immunohistochemical examination. Surgical extirpation is the standard treatment for PMME. Here, we present a case of PMME with unusual endoscopic findings in a Chinese male and review the relevant literature.

## CASE REPORT

**FIGURE 1:**
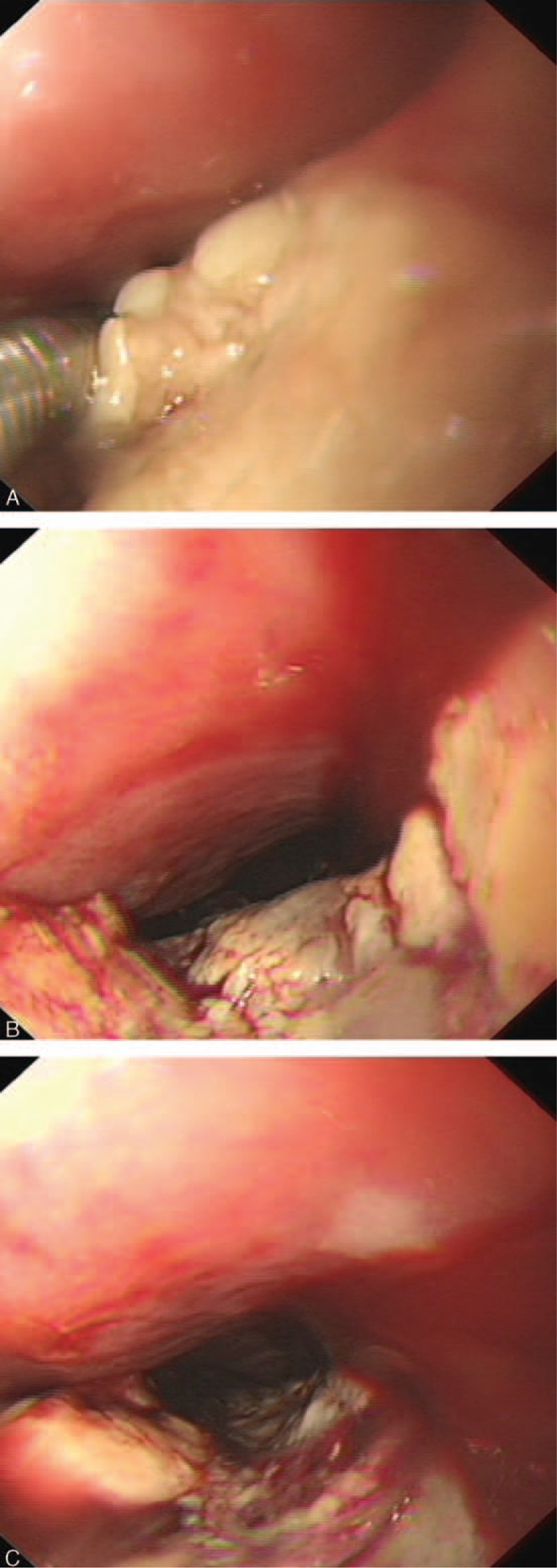
A–C presented the endoscopic images of the primary malignant melanoma of the esophagus from different perspectives. Figure 1 A indicated that there was no pigmentation on the surface. Figure 1 B and C presented that the mass extended progressively for 15 cm along the esophageal longitudinal axis and invaded half of the esophageal circumference. Figure 1 C indicated that the mass had extensive necrotic tissue, active hemorrhage spots and no pigmentation on the surface.

**FIGURE 2:**
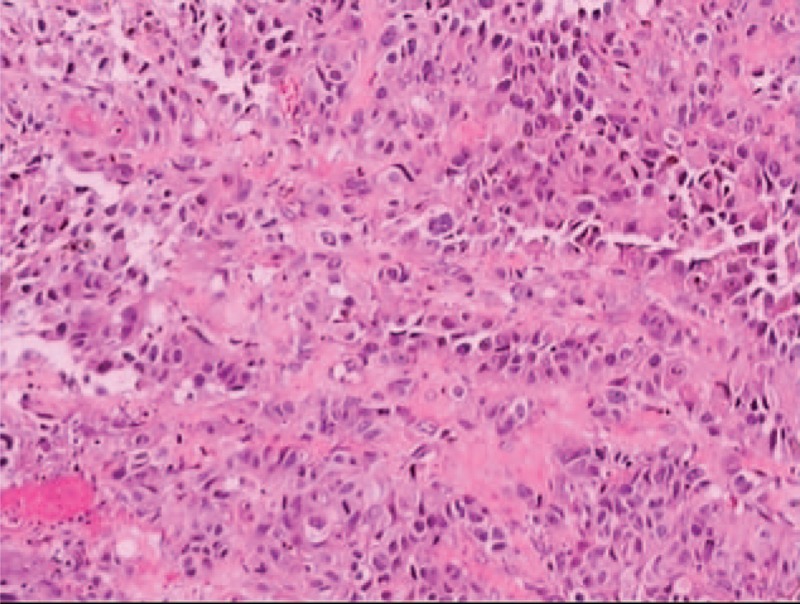
Histopathological staining (hematoxylin and eosin staining) shows the presence of melanin granules.

**FIGURE 3:**
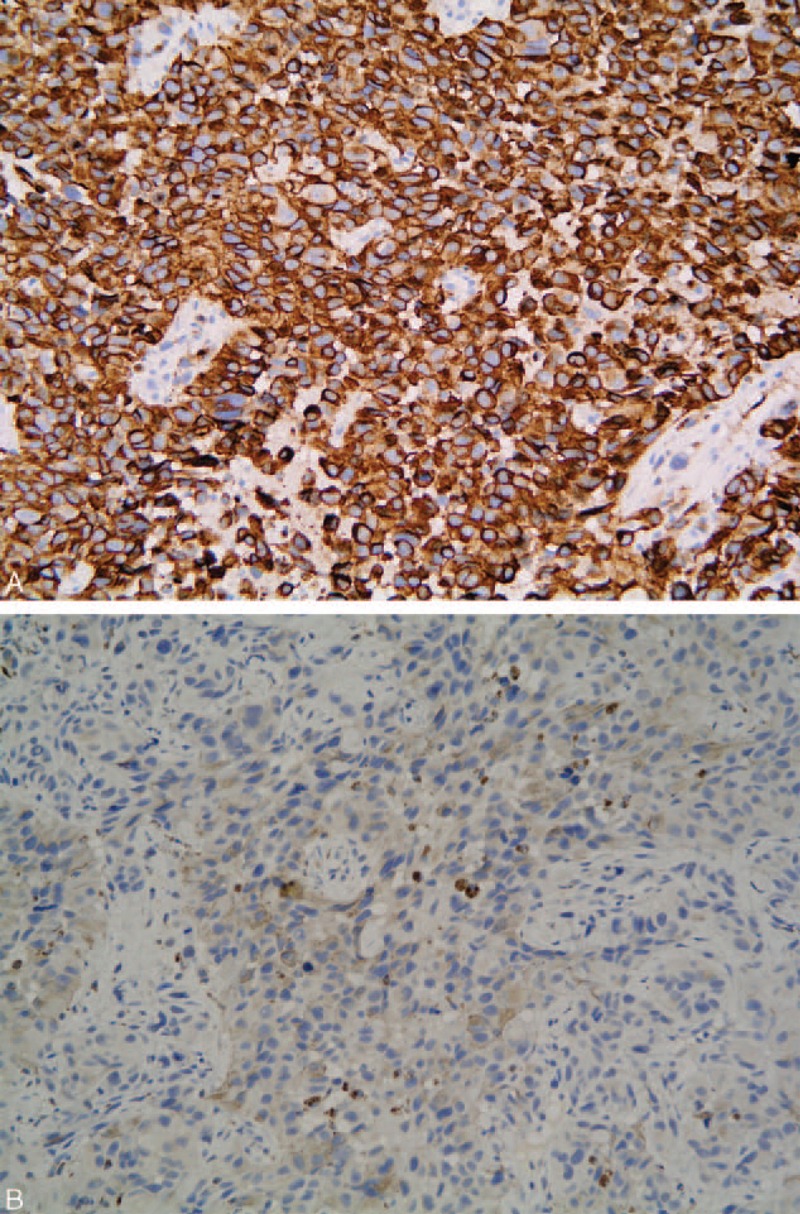
Immunohistochemical staining shows the presence of human melanoma black (HMB)-45 (A) and S100 (B) in the tumor cells. HMB = human melanoma black.

A 79-year-old man was referred to our department because of progressive dysphagia and weight loss for 1 month. Computed tomography performed at a local hospital identified an esophageal tumor; thus, he was admitted to our hospital. He had hematemesis, melena, fever, cough, and yellow sputum. Blood analysis indicated severe anemia and high leukocyte and neutrophil levels. Additionally, the albumin level was very low, and a sputum smear revealed the presence of gram-positive cocci. On endoscopy, an elongated lump was detected on 1 side of the vertical axis of the esophagus approximately 25 to 40 cm away from the incisors. The mass extended progressively for 15 cm along the esophageal longitudinal axis and invaded half of the esophageal circumference. Additionally, the mass had extensive necrotic tissue, active hemorrhage spots, and no pigmentation on the surface (Figure 1A–C). Biopsy was performed, and the tumor was identified as a malignant melanoma (Figure 2). On immunohistochemical staining, the tumor was positive for S-100 and HMB-45 (Figure 3A and B) and negative for cytokeratin and carcinoembryonic antigen markers. Computed tomography showed inflammation of the lungs and no evidence of distant metastasis. Additionally, a physical examination revealed no pigmented lesions at the skin, eyes, rectum, or other locations. Surgery, chemotherapy, or radiotherapy could not be performed owing to the advanced age of the patient and comorbid hypoproteinemia, infection, and severe anemia. Therefore, the patient did not receive treatment and was discharged. After approximately 1 month, we were informed of his death.

## DISCUSSION

PMME is generally considered to be a highly malignant tumor that is associated with a poor prognosis and a rapidly fatal course. The mean patient age at onset is 60.5 years. The incidence of PMME has been reported to be higher in male patients than in female patients, with a ratio of 2:1.^1,2^ The mean survival time from diagnosis has been reported to be 13.4 months.^1,2^ There are 6 reports of patients surviving more than 7 years,^3–8^ and the longest survival time is 12 years.^8^

The tumor is sporadic, and the number of reported cases is small, with only 337 reported cases up to 2011.^9^ In a recently published series of 910 esophageal biopsies in Japan, only 2 were found to be melanoma (0.2%).^10^

The majority of PMME patients present with complaints of dysphagia, nonspecific retrosternal pain, and weight loss. Hematemesis and melena are observed occasionally. A typical finding of PMME is a lobular or polypoid, well-circumscribed, and pigmented tumor. In addition, more than 90% of PMME lesions are located in the distal 2/3 of the esophagus.^11^ The tumor is most often polypoid, and the nonpolypoid form, identified in the present case, is extremely rare. Approximately 10% to 25% of PMME cases present various colors, such as purple, brown, and white, depending on the melanin quantity.^12,13^ The tumor in the present case was 15 cm in length, invaded 1 side of the esophagus, and had no melanin pigmentation on the surface. These findings are not characteristic of PMME. Although tissue pathology identified the presence of melanin granules, the tumor did not have a typical black appearance. We performed immunohistological studies to obtain an accurate diagnosis. On immunohistochemical analysis, the tumor was positive for HMB-45 and S-100. Therefore, we diagnosed the tumor as a malignant melanoma. The diagnosis of PMME is based on the finding of melanin granules in the tumor cells on histological examination, as the clinical and radiological features of PMME are similar to those of other esophageal malignancies. Histological diagnosis can be challenging when the biopsy specimen lacks melanin granules, and the tumor might be incorrectly diagnosed as an epithelial carcinoma. Therefore, when melanin granules cannot be identified in the initial biopsy, immunohistochemical analysis of the biopsy specimen for melanocytic-specific markers, such as HMB-45, S-100, and melanoma-specific antigen (Melan-A), should be performed to obtain an accurate pretreatment diagnosis.

There is a lack of consensus on the treatment of PMME. Cheung et al^14^ found that complete surgical resection was the only treatment strategy that significantly improved survival in PMME patients. Other therapies include chemotherapy, chemoradiotherapy, endocrine therapy, and immunotherapy. However, the roles of these treatment strategies remain unclear. A previous study showed that they might benefit in loco-regional disease control.^15^ Completely nonsurgical treatment strategies have been reported sporadically^16^; however, most of the patients who successfully received these treatments also underwent surgery.^4,16–19^

Molecular analysis could identify targets for additional specific therapy. Mutations in the *BRAF* and *KIT* genes in subsets of melanoma have been reported. Langer et al^20^ detected a mutation of the *c-KIT* gene in 2 patients with PMME. BRAF inhibitors may be useful in the treatment of metastatic melanoma in patients with *BRAF* mutations. However, in patients with PMME, its efficacy has not been proven.^21^

The prognosis of PMME is poor because the tumor has a highly aggressive biological behavior and most patients are diagnosed late. Early diagnosis with complete surgical resection currently offers the best chance of survival.

## CONCLUSION

We reported an extremely rare case of PMME with unusual endoscopic findings. The tumor was diagnosed according to histological and immunohistochemical analyses. Doctors should be aware that PMME might present with unusual endoscopic findings.
